# Sulfite Reductase Co-suppression in Tobacco Reveals Detoxification Mechanisms and Downstream Responses Comparable to Sulfate Starvation

**DOI:** 10.3389/fpls.2018.01423

**Published:** 2018-10-15

**Authors:** Marcel Naumann, Hans-Michael Hubberten, Mutsumi Watanabe, Robert Hänsch, Mark Aurel Schöttler, Rainer Hoefgen

**Affiliations:** ^1^Max Planck Institute of Molecular Plant Physiology, Potsdam, Germany; ^2^Division of Quality of Plant Products, Department of Crop Sciences, University of Göttingen, Göttingen, Germany; ^3^Nara Institute of Science and Technology, Ikoma, Japan; ^4^Department of Plant Biology, Technische Universität Braunschweig, Braunschweig, Germany

**Keywords:** sulfite, sulfide, sulfite reductase, regulation of plant sulfur metabolism, tobacco, co-suppression

## Abstract

Sulfite reductase (SIR) is a key enzyme in higher plants in the assimilatory sulfate reduction pathway. SIR, being exclusively localized in plastids, catalyzes the reduction of sulfite (SO_3_^2−^) to sulfide (S^2−^) and is essential for plant life. We characterized transgenic plants leading to co-suppression of the *SIR* gene in tobacco (*Nicotiana tabacum cv.* Samsun NN). Co-suppression resulted in reduced but not completely extinguished expression of *SIR* and in a reduction of SIR activity to about 20–50% of the activity in control plants. The reduction of SIR activity caused chlorotic and necrotic phenotypes in tobacco leaves, but with varying phenotype strength even among clones and increasing from young to old leaves. In transgenic plants compared to control plants, metabolite levels upstream of SIR accumulated, such as sulfite, sulfate and thiosulfate. The levels of downstream metabolites were reduced, such as cysteine, glutathione (GSH) and methionine. This metabolic signature resembles a sulfate deprivation phenotype as corroborated by the fact that *O*-acetylserine (OAS) accumulated. Further, chlorophyll contents, photosynthetic electron transport, and the contents of carbohydrates such as starch, sucrose, fructose, and glucose were reduced. Amino acid compositions were altered in a complex manner due to the reduction of contents of cysteine, and to some extent methionine. Interestingly, sulfide levels remained constant indicating that sulfide homeostasis is crucial for plant performance and survival. Additionally, this allows concluding that sulfide does not act as a signal in this context to control sulfate uptake and assimilation. The accumulation of upstream compounds hints at detoxification mechanisms and, additionally, a control exerted by the downstream metabolites on the sulfate uptake and assimilation system. Co-suppression lines showed increased sensitivity to additionally imposed stresses probably due to the accumulation of reactive compounds because of insufficient detoxification in combination with reduced GSH levels.

## Introduction

Sulfide (S^2−^) and especially its gaseous form, hydrogen sulfide (H_2_S), have gained increasing interest in terms of their role as regulators or gaseous transmitters in the medicinal field (Tangerman, 2009; Li et al., 2011). Excess amounts are known to produce acute toxicity in humans (Reiffenstein et al., 1992; Truong et al., 2007; Guidotti, 2010) and plants (Baillie et al., 2016). Given the wide variety of its effects in animals, more functions appear to be possible also in plants.

In plants, sulfide is produced by the enzyme sulfite reductase (SIR) as part of the sulfate reduction process during sulfur assimilation or by degradation of cysteine by the enzyme L-cysteine desulfhydrase (DES1) (Álvarez et al., 2010; Romero et al., 2014). H_2_S might function in senescence triggered autophagy (Álvarez et al., 2012) and control of stomatal aperture and, hence, might play a role in drought responses (Jin et al., 2013; Scuffi et al., 2014; Dong et al., 2017; Malcheska et al., 2017).

Sulfite is a Janus-faced molecule. On the one hand, it is a necessary precursor for sulfolipid biosynthesis and for sulfide and cysteine biosynthesis. On the other hand, it has potentially deleterious activities if it accumulates. The chloroplast localized SIR functions as a bottleneck of the sulfate reduction pathway (Khan et al., 2010). It reduces sulfite produced through the activity of APS-reductase (APR) to sulfide, which serves as substrate for *O*-acetylserine(thiol)lyase (OASTL) to synthesize cysteine using the serine derived co-substrate *O*-acetylserine (OAS) (Takahashi et al., 2011). Sulfite is a strong nucleophile; hence, its accumulation would be toxic for the plant as it would randomly attack biomolecules (Pfanz et al., 1987; Nandi et al., 1990) for example resulting in sulfitolysis of proteins (Heber and Hüve, 1997). In consequence, sulfite levels have to be tightly controlled in plants (Hamisch et al., 2012; Randewig et al., 2012; Brychkova et al., 2013). Under certain conditions, such as SO_2_/H_2_S exposure, sulfite levels increase in plants. Sulfite accumulation in cells due to SO_2_ hydration is the reason for the deleterious effects of SO_2_/H_2_S exposition on animals or plants (Murray, 1997; Hänsch et al., 2006; Brychkova et al., 2007). The detoxification of this sulfite proceeds *via* SIR mediated reduction to sulfide and cysteine (Yarmolinsky et al., 2013) and *via* the peroxisomal sulfite oxidase (SO) mediated oxidation to sulfate (Nowak et al., 2004). Sulfate is inert and can re-enter the sulfate assimilation pathway or can be stored in the vacuole (Kaiser et al., 1989; Martinoia et al., 2000).

SO_2_/H_2_S exposition is deleterious for not only plants (Baillie et al., 2016) but also humans (Sang et al., 2010). However, it has been reported that SO_2_ is actively produced in human tissues to provide a signaling or health promoting activity (Liang et al., 2011). Therefore, it might be necessary to readdress the function of SO_2_/sulfite in plant tissues. Han et al. (2018) showed that external sulfite provision might play a role in cadmium tolerance, not only through the additional sulfur supply resulting in increased concentration of the redox regulating tripeptide glutathione (GSH) and probably increased phytochelatin levels but also through specific transcriptional changes of the cadmium uptake and antioxidant systems. Further, a role of exogenously supplied SO_2_ in pathogen defense might be considered (Xue and Yi, 2018). As SO produces one molecule of H_2_O_2_ per oxidized molecule of sulfite, excessive sulfite levels might lead to an accumulation of H_2_O_2_, further contributing to the damage, despite the fact that some non-enzymatic conversion of sulfite to sulfate with H_2_O_2_ as a reaction partner does occur (Hänsch et al., 2006). Both, SO_2_ and SO_2_ detoxification derived H_2_O_2_, might contribute to damage and to signaling (Dat et al., 2000). These anti-pathogenic and signaling functions might make it necessary to reconsider the role of endogenous sulfite in plant defense (Tahir et al., 2013) and plant metabolism. A possible association of sulfate metabolism in response to sulfate deprivation and responses to increased contents of H_2_O_2_ has been suggested with respect to the induction of a subgroup of sulfate response genes, termed OAS-cluster genes (Hubberten et al., 2012b).

Sulfide production by SIR in photosynthetic tissues is dependent on ferredoxins reduced by photosystem I or by a NADPH dependent ferredoxin-NADP^+^-reductase (FNR; EC 1.18.1.2) to provide six necessary electrons for sulfite reduction (Yonekura-Sakakibara et al., 1998; Akashi et al., 1999; Nakayama et al., 2000; Yonekura-Sakakibara et al., 2000). Sulfide produced by SIR in the chloroplast has been shown to be available for cysteine biosynthesis in all three compartments – chloroplasts, mitochondria, and cytosol (Heeg et al., 2008; Watanabe et al., 2008b; Krueger et al., 2009; Hell and Wirtz, 2011). Hence, sulfide is able to permeate all subcellular membrane systems. Cysteine is the common precursor of a vast variety of downstream compounds containing organic sulfur, including the amino acid methionine, GSH, sulfolipids, vitamins, and co-factors to numerous secondary compounds (Takahashi et al., 2011). Controlled flux and balance of the sulfur assimilation pathway implies a controlled sulfite/sulfide junction to assure physiological integrity of plants. This has been shown by downregulating the enzymatic activity of SIR in Arabidopsis (Khan et al., 2010; Wang et al., 2016) and tomato plants (Yarmolinsky et al., 2014).

In order to investigate the consequences of a blockage of sulfide production at the metabolic level, we have employed tobacco plants (*Nicotiana tabacum* cv. Samsun NN) exhibiting reduced SIR activity due to co-suppression (Lein et al., 2008). The metabolic consequences for the plants with respect to metabolism upstream and downstream of the SIR interface were investigated. SIR activity was reduced to about 20–50% of activity in wild-type plants (WT). Sulfite levels were increased due to the reduction of SIR activity and at the same time sulfate and thiosulfate contents accumulated in leaf tissues. We interpret this as the combined effect of detoxification mechanisms and downstream signals. Sulfide levels remained constant, but metabolites downstream of sulfide in the *SIR* co-suppression lines displayed a sulfur starvation phenotype, namely cysteine and GSH contents were reduced while OAS accumulated. These downstream alterations obviously act as signals to override sulfate uptake control as sulfate levels increased. The co-suppression lines showed increased sensitivity to additionally imposed stresses. With respect to sulfide, this allows to draw the conclusions that sulfide homeostasis is crucial for plant performance and survival, but that sulfide does not act as a signal in this context to control sulfate uptake and assimilation.

## Results

### Phenotypic Effects of SIR Co-suppression on Tobacco

**FIGURE 1:**
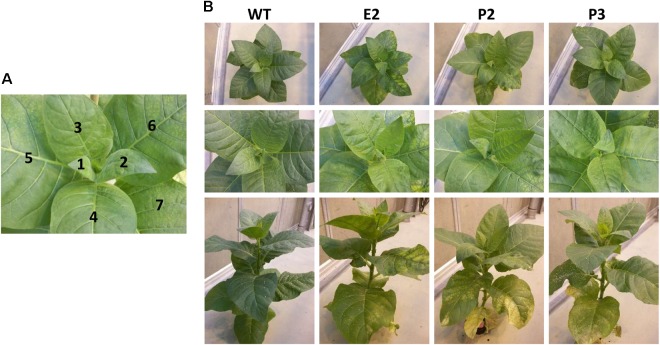
Sulfite reductase (SIR) co-suppression tobacco lines. **(A)** Leaf numbering indicates the consecutive numbering of leaf samples for harvesting from youngest leaf (L1) to oldest leaf harvested (L7). **(B)** Tobacco plants at the harvesting stage (approximately 2-month-old plant). Each panel shows a representative plant. Starting with L5 leaf chlorosis becomes visible. The phenotype actually resembles that of sulfate starvation as around vasculature tissue remains greener than between veins. In some cases, a retarded growth phenotype was observed. This P2 plant displays strong chlorosis and necrosis associated with older leaves.

For investigating the effect of SIR co-suppression, we have chosen three mutant lines from the original large-scale phenotyping experiment aimed at identifying genes essential for leaf function. In total, 20,000 randomly chosen genes were transferred into tobacco as co-suppression or antisense constructs (Lein et al., 2008). First pairwise (P) transformations of two independent genes was performed and after identification of leaf chlorotic or necrotic leaf phenotypes, single constructs (E) were transformed into tobacco (*Nicotiana tabacum cv.* Samsun NN). Among the 77 identified essential genes, one was determined to be a tobacco SIR. The sequence used for the transformation is shown in **Supplementary Figure 1**. The plants chosen are construct numbers E-18042 (termed E2), P-14921 (termed P2), and P-14921-2_3a (termed P3). These plants and their subsequent generations showed variability of the phenotype. The plants generally appeared to be slightly paler (chlorotic) than controls. Older leaves displayed chlorotic patches or spots and some even developed local necrosis. Young leaves (sink leaves) displayed no or weak chlorotic phenotypes while chlorotic or necrotic phenotypes occurred in an increasing gradient from young to old leaves (**Figure 1**). Some individual plants of E2, P2, or P3 displayed retarded growth and stronger phenotypes. The occurrence of these phenotypic patterns could not be assigned to any obvious growth conditions and seemed to appear randomly.

### Enzyme Activity and *SIR* Expression

**FIGURE 2:**
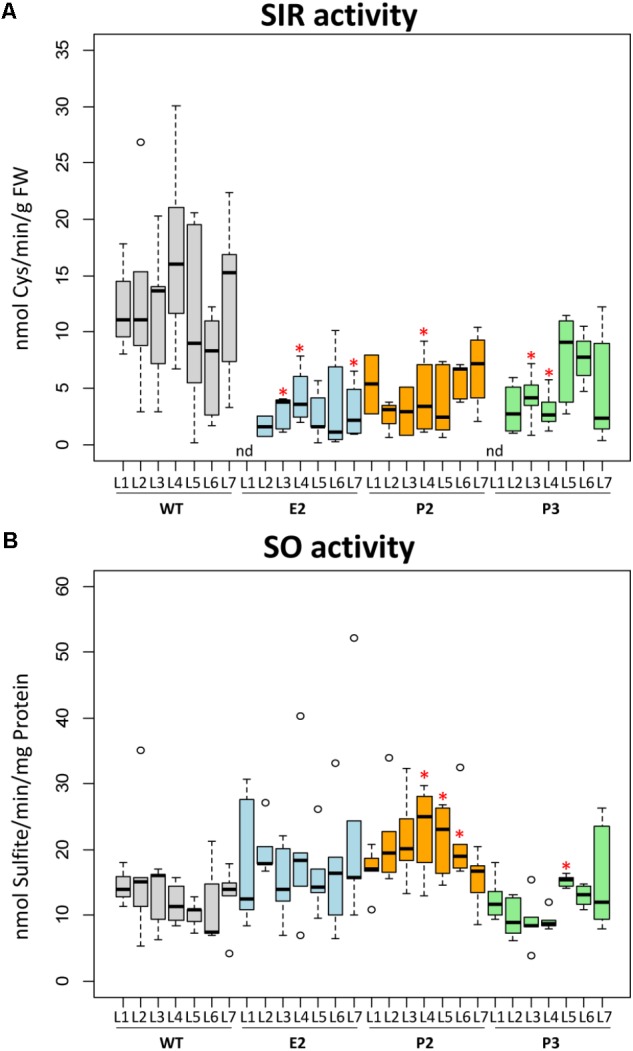
SIR and SO activity. Boxplots show enzymatic activities of sulfite reductase (SIR) **(A)** and sulfite oxidase (SO) **(B)**. The top edge (hinge) of the box indicates the 75th percentile and the bottom hinge indicates the 25th percentile of data. The line in the box indicates the median value. The ends of the vertical lines (whiskers) indicate the minimum and maximum data values. Outliers are represented by an open circle. Differences between the wild-type and transgenic lines were analyzed using Student’s *t*-test and statistical significance was indicated (^∗^*P* < 0.05). nd, not determined because below detection limit.

Expression of the *SIR* gene of tobacco (*NtSIR*) was reduced to about 20–30% (E2 lines) of WT plants (**Supplementary Figures 2A,B**). We determined the enzyme activities of SIR and SO in leaf samples of wild-type (WT) control plants and lines E2, P2, and P3. Samples were taken from top (L1) to bottom (fully expanded) leaves (L7). SIR activity showed high variation in the leaves of control plants with no apparent pattern. SIR activity was reduced in the co-suppression lines E2, P2, and P3 compared to WT plants to about 20–50%. Due to the high variation observed in WT samples, the reduction of SIR enzyme activity in transgenic plants was rendered insignificant for most samples with best confidence for E2 (**Figure 2A**). The medians, especially of E2 and P2, are clearly below WT levels. As we assumed that sulfite should accumulate due to this reduced SIR activity, we also determined the enzyme activity of SO. SO detoxifies sulfite and is induced under conditions where sulfite accumulates such as SO_2_ exposition (Hänsch et al., 2006). SO activity displayed a tendency for slight increasement in the lines E2 and P2 but not P3 in comparison to controls (**Figure 2B**).

### How Does SIR Co-suppression Affect the Composition of the Sulfate Assimilation Pathway?

**FIGURE 3:**
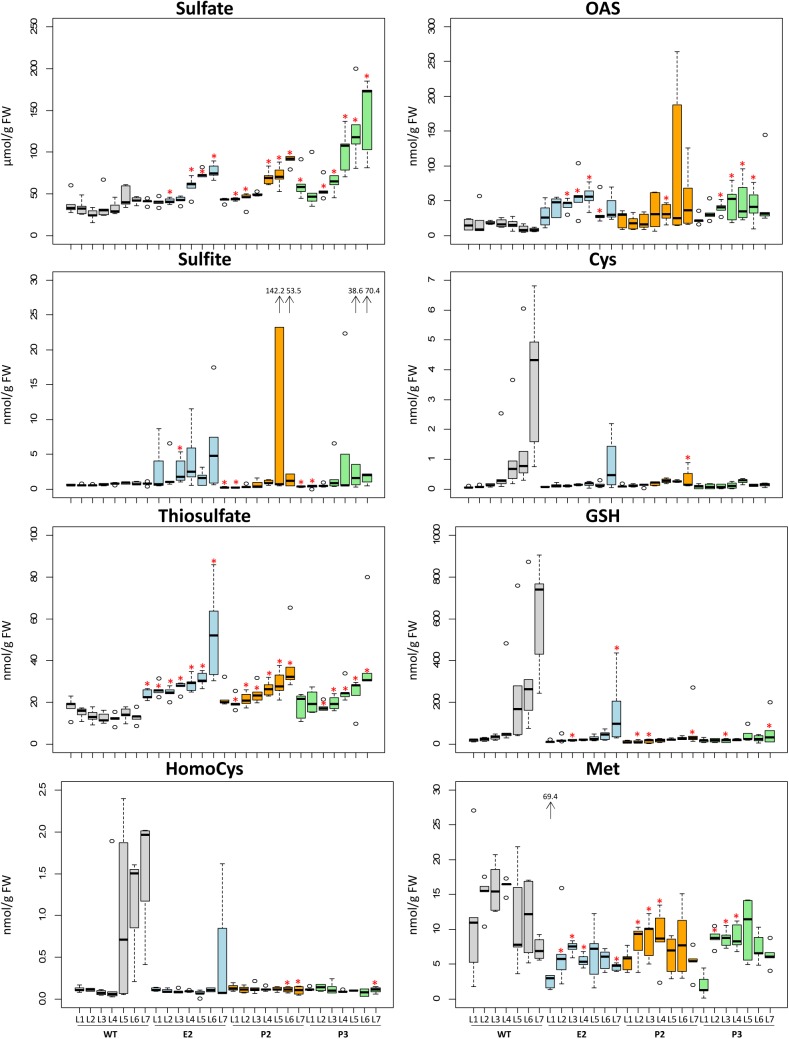
Changes of contents of metabolites related to sulfur metabolism. Boxplots show metabolite changes in sulfur metabolism. Details of boxplot interpretation are given in **Figure 2**. Extreme outliers were shown with arrows and the values. Differences between the wild-type and transgenic lines were analyzed using Student’s *t*-test and statistical significance was indicated (^∗^*P* < 0.05).

**FIGURE 4:**
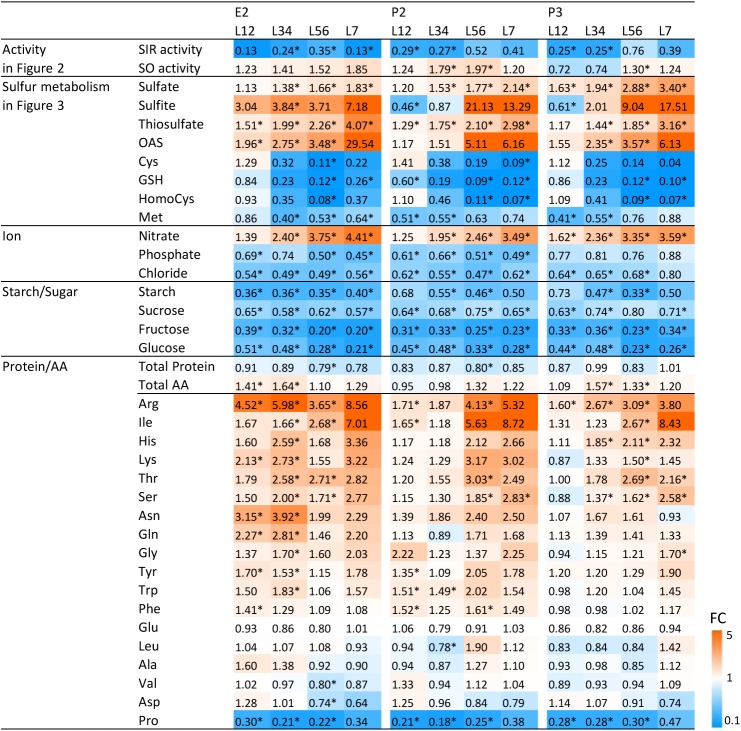
Changes of SIR and SO activities and metabolites in the transgenic lines compared to wild-type plant. Contents of amino acids and thiols were determined by HPLC analysis and ions by ion chromatography. Contents of starch, sugars, total protein, total amino acid, and proline were determined by a 96-well platform (see materials and methods). The previously shown data (**Figures 2**, **3**) were included. The ratios of fold changes from wild-type plants are given by shades of red or blue colors according to the scale bar. In order to generalize the datasets, we averaged sample results and fused the data pairwise from young to old leaves, i.e., L12 (L1 + L2), L34 (L3 + L4), L56 (L5 + L6), and L7. Data represent the mean (±SD) of 10 biological replicates for L12, L34, and L56, and five for L7. Differences between the wild-type and transgenic lines were analyzed using Student’s *t*-test and statistical significance was indicated (^∗^*P* < 0.05). FC, fold-change.

In the SIR co-suppression lines E2, P2, and P3 metabolites of the primary sulfate metabolism were determined in order to investigate for the effect of the introduced bottleneck in the primary sulfur assimilation pathway (**Figures 3**, **4**). Free ionic sulfate levels increased significantly in most samples in comparison to WT. Increases were insignificant only in young, i.e., sink leaves, probably provided by sulfate transport from mature leaves of E2, P2, and P3. Sulfate levels in tobacco control plants (WT) were strictly controlled between about 25 and 50 μmol/g FW with slight increases toward older leaves. In lines E2, P2, and P3 this control is abrogated as sulfate accumulates with increasing leaf age, reaching between 75 and 100 μmol/g FW in E2 and P2, and more than 150 μmol/g FW in P3. Free sulfite levels were hardly detectable in leaves of WT plants, while the reduction of SIR activity resulted in increases of sulfite contents mainly in mature leaves of E2, P2, and P3. Single values for older leaves of P2 and P3 were extremely high. Thiosulfate significantly increased in all three transgenic lines and increased from young to old leaves in a pattern paralleling sulfate accumulation. Thiosulfate levels in WT remained low in all leaves and rather displayed an opposing trend in older leaves showing slightly decreased contents. With respect to the sulfur amino acids, cysteine and GSH levels increasingly accumulated with leaf age in WT plants. In E2, P2, and P3 this increase with leaf age is reduced as the reduced SIR activity can be assumed to result in a reduced flux to sulfide for cysteine biosynthesis. This pattern resembles that of sulfate starvation, despite of the fact that the transgenic plants over-accumulate sulfate. This conclusion is supported by the fact that OAS as the second precursor of cysteine biosynthesis next to sulfide tends to accumulate in E2, P2, and P3, though the increases were not as high as under sulfate starvation conditions. It might be speculated that the remaining SIR activity is just sufficient to provide sulfide for downstream synthesis of sulfur compounds to facilitate survival. From the methionine branch, the contents of the methionine precursor homocysteine and of methionine were determined. Homocysteine levels increased in leaves of mature WT tobacco plants (L5, L6, and L7). In E2, P2, and P3, homocysteine levels were comparably low in young leaves and remained low in older leaves (apart from L7 of E2) displaying clear tendencies when comparing the medians. Methionine levels varied quite substantially and with no obvious pattern in WT leaves. The transgenic lines displayed reduced contents of methionine, especially in younger leaves.

Sulfide levels were determined in a later experiment in descendants of E2, the single *SIR* co-suppression line, using lines termed E2-2b, E2-3a, and E2-3b (**Supplementary Figures 2A,C**). As before, lines displayed variable phenotypes from almost WT-like appearance with slight chlorotic patches in older leaves of E2-3a toward visible phenotypes (**Supplementary Figure 2A**). E2-2b showed chlorosis and necrosis only in older leaves, while E2-3b displayed a chlorotic phenotype all over and reduced growth resembling sulfate starvation with darker green tissues around the vasculature and pale intercostal fields (**Supplementary Figure 2A**). Descendants of E2-3b again either displayed a visible phenotype all over (E2-3b-1) or a slighter phenotype (E2-3b-2). No significant differences between sulfide levels were detected in plants and leaf stages of E2-2b, E2-3a, and E2-3b. Leaves were sampled from top to bottom, young to old (depicted as L1 to L4). Sulfide contents, at 25–30 nmol/g FW, were much higher in tobacco than reported for example for tomato (3–6 nmol/g FW; Yarmolinsky et al., 2014), Arabidopsis (approximately 10 nmol/g FW; Hubberten et al., 2012b). This puts an additional pressure on the transgenic plant lines as tobacco seems to have a higher need for available sulfide.

In summary, a picture emerges that sulfate, as an upstream component relative to the SIR bottleneck, accumulates. In parallel, thiosulfate accumulates, which is normally hardly detectable in tobacco leaves. Sulfite levels showed a tendency to increase, especially with increasing leaf age. Metabolites downstream of SIR displayed in *SIR* co-suppression lines a pattern resembling sulfate deprivation responses (Nikiforova et al., 2005) while sulfide levels remained stable despite reduction of SIR activity.

### Metabolic Analysis of Secondary Effects

As the bottleneck introduced by reduction of SIR activity provoked changes in the metabolic composition of primary sulfur assimilation pathway, we investigated whether further pleiotropic effects were generated. We determined the content of the nutrient ions phosphate and nitrate additionally to sulfate, the carbohydrates starch, sucrose, fructose, and glucose, total protein contents, total amino acid contents, and the proteinogenic amino acids in WT plants and the transgenic lines E2, P2, and P3. Data were plotted as ratio of the respective metabolite contents of the transgenic lines toward WT control and displayed as a heatmap (**Figure 4**). In order to generalize the datasets and to identify trends, we averaged sample results and fused the data pairwise from young to old leaves—i.e., L1 + L2, L3 + L4, L5 + L6, and L7. The previously shown data (**Figures 2**, **3**) were included in the heatmap applying the same grouping.

Data displayed the same patterns (**Figure 4**) for all three transgenic lines as described above (**Figures 2**, **3**). SIR activity is reduced and SO activity is mildly increased with a preference for older leaves. Precursors upstream of the sulfite/sulfide bottleneck, namely sulfate, sulfite, and thiosulfate, are increased – again with a preference to be increased with increasing leaf age. Metabolites downstream of the sulfite/sulfide bottleneck, namely Cys, GSH, and Met are reduced while the co-substrate of sulfide for Cys formation, OAS, was increased. Nitrate contents increased, while phosphate and chloride contents were reduced.

Despite sufficient nitrogen and sulfur availability and only moderate phosphate decrease, total protein amounts displayed a tendency toward reduction in E2, P2, and P3 when compared to WT. At the same time, total free amino acid levels increased moderately. When determining the accumulation patterns of single amino acids, the results were more complex. From the 20 proteinogenic amino acids, methionine and cysteine were reduced in E2, P2, and P3, as shown in **Figure 3**. Younger and therefore sink leaves (L1-L2) even showed increases in cysteine content. All other amino acids besides the pyruvate derived amino acids Leu, Ala, Val and the amide amino acid precursors glutamate and aspartate displayed a tendency to be increased, especially in older leaves. The N rich amino acids arginine, lysine, glutamine, and asparagine seemed to accumulate in response to excess nitrate availability or insufficient amino acid conversion to proteins. Proline was consistently low in all three transgenic lines (E2, P2, and P3) compared to WT.

### Chlorophyll Content and Photosynthesis

**FIGURE 5:**
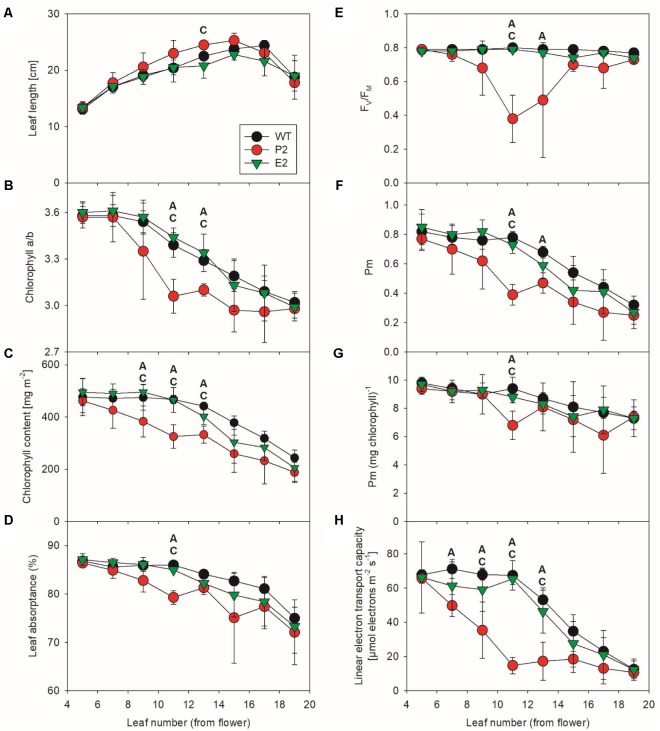
Photosynthetic function in SIR co-suppression lines. Changes in leaf length **(A)**, chlorophyll a/b ratio **(B)**, total chlorophyll content per leaf area **(C)**, leaf absorptance **(D)**, the maximum quantum efficiency of PSII in the dark-adapted state (F_V_/F_M_, **E**), the amount of redox-active PSI per leaf area **(F)** and per chlorophyll **(G)**, and in linear electron transport capacity **(H)** during leaf ontogenesis in tobacco. For each time point of the developmental series, an ANOVA was performed for the wild-type, the strongest (P2) and the weakest (E2) mutant line. Statistically significant differences between wild-type and P2 (indicated as “A”), wild-type and E2 (indicated as “B”), and between the two co-suppression lines (indicated as “C”) are shown (*P* < 0.05).

To characterize effects of the co-suppression lines on photosynthesis, we analyzed fully developed greenhouse-cultivated plants at the onset of flowering. We selected two mutant lines showing the strongest and weakest phenotype, respectively, under these growth conditions. Thus, we covered a wide range of potential photosynthetic defects (**Figure 5**). While the weakest line E2 only displayed minor patchiness at the edges of some mature leaves, the mature leaves of the most strongly affected line P2 contained leaf sectors, which were completely bleached and necrotic. Because these sectors were not measurable with our spectroscopic techniques, the severity of the photosynthetic defects of the strongly affected co-suppression line might be underestimated in this experiment. To obtain a detailed overview of possible leaf-age related photosynthetic defects in the mutants, eight different leaf generations were measured, starting with the youngest leaf of more than 10 cm leaf length, which usually was the fifth leaf below the flower (**Figure 5A**). Then, each other leaf was measured, down to the visibly senescent leaf number 19. We failed to observe significant differences for any photosynthetic parameter between the young leaves number five and seven of the WT and the co-suppression lines. However, in accordance with the visual phenotype of the two co-suppression lines, with increasing leaf age, we observed several significant differences between the WT and the strong mutant line P2 (indicated by “A” in the **Figure 5**). This was most often the case in the middle leaf generations, which were fully expanded at the time of the experiment. In the oldest leaf generations, differences became less pronounced, probably due to increasing senescence of the older leaves also in WT. We failed to observe a significant difference between the WT and the weak mutant line E2 (which would have been indicated by “B”). However, in several cases, lines E2 and P2 were also significantly different from each other (indicated by “C” in **Figure 5**), but this was less often the case than significant differences between P2 and the WT, in line with the fact that E2 behaved in an intermediate way between WT and the strong co-suppression line.

Leaf length (**Figure 5A**) increased for the WT and both co-suppression lines with leaf age, except for the oldest leaf, which had already become senescent and started to loose turgor. The chlorophyll a/b ratio (**Figure 5B**) decreased slowly with increasing leaf age in the WT and the weak mutant line E2. This can be explained as a typical ontogenetic decrease in PSII reaction center content and a parallel increase in LHCII abundance, resulting in an increased antenna size per PSII reaction center. This has been previously reported for tobacco (Schöttler et al., 2004, 2007, 2017) and many other species and is attributable to older leaves getting progressively shaded (reviewed by Schöttler and Toth, 2014). However, in the strong mutant line P2, this leaf-age dependent decrease in the chlorophyll a/b ratio was strongly accelerated, and also total chlorophyll content per leaf area (**Figure 5C**) decreased more rapidly with leaf age than in the WT and the weak mutant line. This suggests an accelerated loss of photosynthetic reaction centers, which bind only chlorophyll a, in the strong mutant line.

In line with this assumption, we found clear indications for photodamage to PSII. The maximum quantum efficiency of PSII photochemistry in the dark-adapted state (F_V_/F_M_, **Figure 5E**) is a measure for the functional integrity of PSII. It was drastically decreased in the middle leaf generations of the strong co-suppression line, but recovered again in the older leaves. Possibly, these old leaves are so heavily shaded by younger leaves that the light intensity reaching them is too low to induce PSII photoinhibition. Also Pm, a measure for the amount of redox-active PSI per leaf area, decreased more rapidly in the strong mutant line than in the WT and line E2 (**Figure 5F**). For the WT and the weak mutant line, the observed leaf-age related decrease in Pm per leaf area is well in line with typical leaf age–related decreases of PSI content, which usually strictly parallel changes in the abundance of the entire photosynthetic apparatus and in chlorophyll (reviewed by Schöttler and Toth, 2014). Therefore, on a chlorophyll basis, which is a good proxy for the composition of the photosynthetic apparatus per thylakoid membrane, PSI content is usually very stable and largely independent of leaf ontogenesis or changing environmental conditions (Schöttler and Toth, 2014). To test if this is also the case here, we re-normalized Pm to the chlorophyll content of the leaf (**Figure 5G**). After this re-normalization, both differences between lines and age-related differences became less pronounced. Therefore, the accelerated loss of PSI in the middle leaf generations of the strong mutant line is unlikely to be due to specific damage to PSI and its oxidative destruction. Instead, because chlorophyll and PSI decrease in parallel, an accelerated but controlled degradation of the entire photosynthetic apparatus with increasing leaf age seems to occur, possibly due to an accelerated leaf senescence program.

Finally, we measured light response curves of linear electron transport by chlorophyll-a fluorescence-based determinations of the photochemical yield of PSII (**Figure 5H**). The calculated electron transport capacity in saturating light was corrected for differences in leaf absorptance of photosynthetically active radiation (**Figure 5D**). This was necessary because due to its accelerated loss of chlorophyll, the photosynthetic apparatus of the leaves of the strong mutant absorbed less excitation energy to drive electron transport. For the WT and the weak mutant, we observed the typical leaf-age related decline in photosynthetic electron transport, which started from leaf number 11 onward and has been previously reported multiple times for tobacco leaf ontogenesis (Schöttler et al., 2004, 2007, 2017). However, in the strong co-suppression line, this repression in electron transport capacity was strongly accelerated, in line with the scenario of an early onset of a leaf senescence program. Down-regulation of linear electron transport progressed even faster than the appearance of photoinhibited PSII and the decrease in PSI and total chlorophyll per leaf area. Therefore, it seems likely that other components of the electron transport chain, such as cytochrome b_6_f complex and plastocyanin, which usually control linear electron transport in tobacco (Schöttler et al., 2004, 2007), are also rapidly repressed in the co-suppression line, but this needs to be addressed in a more detailed separate analysis.

### Does Additional Stress Affect SIR Co-suppression Lines?

**FIGURE 6:**
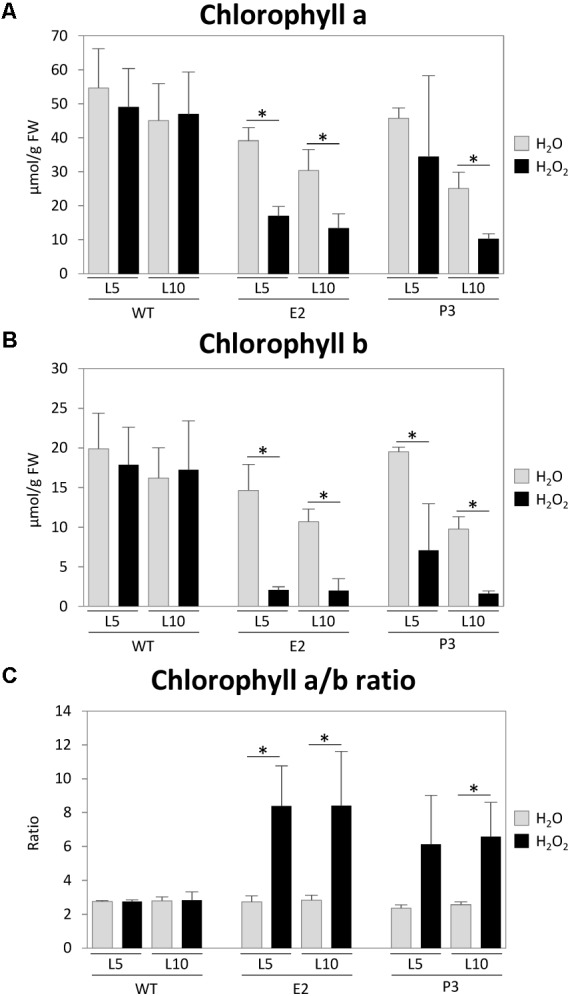
H_2_O_2_ treatment experiment. Small leaf disks from leaves (L5 and L10) were treated with a 1 M H_2_O_2_ solution for 3 days. Chlorophyll contents **(A,B)** were measured and the ratio **(C)** was determined and compared with those of water treated plants. Data represent the mean (±SD) of three biological replicates. Differences between the H_2_O_2_/water treated plants were analyzed using Student’s *t*-test and statistical significance was indicated (^∗^*P* < 0.05).

Sulfite reductase inhibition and hence accumulation of sulfite, coupled with insufficient sulfide supply and therefore reduction of downstream metabolite contents including GSH, should lead to a reduced capacity of the cell to deal with additionally imposed stresses caused by reactive oxygen species. This higher sensitivity has previously been shown for Arabidopsis SIR RNAi lines using methylviologen to induce ROS production (Wang et al., 2016). We exposed leaf disks of leaf 5 (L5) and leaf 10 (L10) of WT tobacco plants and lines E2 and P2 to 1 M H_2_O_2_ in 6-well petri dishes. The stress response was determined by measuring the relative reduction of chlorophyll content (Willekens et al., 1997; Watanabe et al., 2010) (**Figure 6**). While the chosen concentration of H_2_O_2_ had no significant effect on WT chlorophyll contents, chlorophyll a and b contents were reduced in the transgenic lines, especially in the older leaf (L10). This is indicative of an already existing stress level in the transgenic lines and therefore a reduced capacity to deal with ROS, which is in line with the observed PSII photoinhibition in mature leaves of greenhouse-grown plants of the strong co-suppression line under standard conditions (**Figure 5E**). It has been previously shown that plants impaired in SIR activity cannot compensate for additional stresses, which results in increased chlorophyll degradation. This has been shown for sulfite injection into tomato plant leaves where the chloroplast localized SIR is downregulated by extended darkness (Brychkova et al., 2013) or methylviologen treatment of Arabidopsis SIR RNAi lines (Wang et al., 2016). The ratio of chlorophyll a/b is usually about 3:1 as in WT and transgenic lines (P2 and P3) under mock treatment (water). In WT plants, the H_2_O_2_ treatment did not lead to a change of the chlorophyll ratio, whereas the ratio shifted to 8:1 for P2 lines and to 6:1 for P3 lines, irrespective of leaf age. Such increases in the chlorophyll a/b ratios (**Figure 6**) hint at ROS induced reduction of the antenna system of PS II (Strand et al., 2015), which usually occurs in response to high-light conditions resulting in increased singlet oxygen production in PSII. It could be also indicative of nutrient depletion induced senescence (NUDIS) (Pruzinská et al., 2005; Watanabe et al., 2010, 2013).

## Discussion

Studies on plants with altered *SIR* expression and hence activity have been performed previously using T-DNA knock-down mutants in Arabidopsis (Khan et al., 2010) and *SIR* RNAi mutants in both, tomato (*Solanum lycopersicon*) (Yarmolinsky et al., 2014) and Arabidopsis (Wang et al., 2016). Additionally, extended exposure to darkness led to reduced SIR activity in tomato (Brychkova et al., 2013). Furthermore, overexpressing a *Pseudomonas areoginosa APR* in *Arabidopsis thaliana* and *Zea mays* affected the balance of the sulfide/sulfite junction leading to elevated contents of the upstream compounds sulfite and thiosulfate, and of the downstream metabolites cysteine, γ-glutamylcysteine, and GSH, which indicated an insufficient provision of OAS (Tsakraklides et al., 2002; Martin et al., 2005).

In this study, we have used *SIR* co-suppression in tobacco to probe the sulfite/sulfide junction in plant cells. These co-suppression mutants were obtained from a large-scale screen for genes, which play a critical role for leaf development, metabolism, and especially photosynthesis. To this end, a tobacco leaf cDNA library covering more than 20000 genes was cloned into co-suppression vectors, and then transformed into WT tobacco. Then, mutants were screened for visual defects in leaf functions, such as chlorotic or necrotic leaves (Lein et al., 2008). Because co-suppression in tobacco usually results in much weaker reductions in mRNA levels than RNAi approaches, only such mutants should display a phenotype, whose target genes are especially sensitive already to a partial down-regulation and therefore likely play a rate-limiting role in their pathway (Lein et al., 2008). In total, this screen led to the identification of 88 such critical genes, with functions mainly in photosynthetic light reactions and chorophyll biosynthesis. Also genes involved in protein targeting and degradation were strongly enriched. Finally, several genes of previously unknown function were identified, which since then were ascribed functions in photosystem I assembly (Y3IP1; Albus et al., 2010) and chlorophyll biosynthesis (LCAA; Albus et al., 2012). The *SIR* co-suppression mutant was the only identified mutant involved in sulfur metabolism visibly affecting chlorophyll accumulation, suggesting that SIR plays a central role in tobacco leaf sulfur metabolism, or that a de-regulation of this reaction in the pathway results in metabolic disturbances and the accumulation of toxic intermediates affecting the photosynthetic apparatus.

Indeed, SIR is an essential step in sulfur assimilation and both, its educt sulfite and product sulfide act as precursors in the sulfur assimilation pathway and presumably as signaling molecules affecting enzyme activities and gene expression within the pathway (Hoefgen and Hesse, 2008; Álvarez et al., 2012; Yarmolinsky et al., 2013). Further, SIR plays a role in the detoxification of endogenous catabolic sulfite from protein or sulfolipid degradation or exogenous SO_2_ exposition (Hänsch et al., 2006; Yarmolinsky et al., 2013).

**FIGURE 7:**
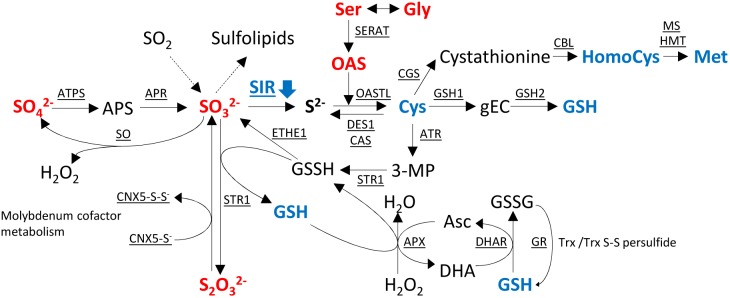
Changes of metabolites in sulfur metabolism in the transgenic *SIR* co-suppression lines compared to wild-type plants. Metabolites in red indicate increased levels and metabolites in blue indicate decreased levels of the metabolite in the transgenic lines compared to wild-type plants. APS, 5′-adenosine phosphosulfate; gEC, γ-glutamylcysteine; GSH, glutathione; GSSH, glutathione disulfide; OAS, *O*-acetyl serine; 3-MP, 3-mercaptopyruvate; Asc, ascorbate; DHA, dehydroascorbate; ATPS, adenosine triphosphate sulfurylase; APR, APS reductase; SIR, sulfite reductase; SERAT, serine acetyltransferase; OASTL, *O*-acetylserine(thiol)lyase; DES1, cysteine desulfhydrase 1; CAS, cyano-alanine synthase; GSH1, γ-L-glutamyl cysteine synthetase; GSH2, glutathione synthetase; CGS, cystathionine γ-synthase; CBL, cystathionine β-lyase; MS, methionine synthase; HMT, homocysteine *S*-methyltransferase; ATR, aminotransferase; STR1, sulfurtransferase; ETHE1, ethylmalonic encephalopathy protein 1; APX, ascorbate peroxidase; DHAR, dehydroascorbate reductase; GR, glutathione reductase; SO, sulfite oxidase; Trx, thioredoxin; CNX, cofactor for nitrate reductase and xanthine dehydrogenase.

As sulfite is a precursor of various linked pathways (**Figure 7**), alteration of SIR activity resulted in distinct changes, which have been described in the aforementioned publications and which are corroborated through this study in tobacco *SIR* co-suppression lines. The strongest phenotypes have been described for *sir1-1* knock-down lines which were seedling lethal in the homozygous state and pale dwarfs as heterozygous plants (Khan et al., 2010). Tomato RNAi lines, as the tobacco co-suppression lines of this study, displayed a patchy phenotype with chlorosis and necrosis (Yarmolinsky et al., 2013, 2014). Tobacco co-suppression lines of this study displayed dwarfism in an unpredictable manner. In particular, we could not relate the high variation of SIR activity in control plant leaves to any obvious conditions of the greenhouse grown plants. High variation between samples seems to be a specific feature of the tobacco co-suppression lines. Variability was not a technical feature of the samples, as other parameters measured from the very same tissue samples displayed much less variation. A possible explanation might be that the knock-down of the essential SIR enzyme would be lethal for tobacco throughout the selection procedure during tissue culture when exceeding a certain level of inhibition. As such, only plants with a mild phenotype due to a partial reduction would survive. Variability of the visible phenotypes and patchiness in leaves might derive from the fact that the deleterious effect is only reached when a threshold has been passed in the tissue. It has been shown that diverse tissues display differential expression patterns in various Arabidopsis tissues (Wang et al., 2016) and that SIR activity displays a diurnal pattern with reduced night activity (Brychkova et al., 2013). Actually, because of the highly variable SIR activity in WT, SIR activity might fall under a critical threshold in certain tissue parts but not others, thus resulting in the observed patchiness. Further, variability of the phenotype might well be the effect of epigenetic control as tobacco tends to counteract deleterious transgenes (Hoefgen et al., 1994). The high sulfide levels in tobacco (**Supplementary Figure 2C**) might lead to an additional selection of plants with mild phenotypes, i.e., plants with a remaining SIR activity matching the comparably high sulfide needs of tobacco and its capacity for detoxification of the accumulating sulfite. Additional stresses, such as a reduction in the GSH-ascorbate cycle (**Figure 7**), paired with local developmental or externally imposed imbalances, might trigger tissue damage through ROS, probably through H_2_O_2_ accumulation (Willekens et al., 1997).

Despite this variability, the down-regulation of *SIR* expression and activity results in the reduction of metabolites downstream of the sulfite/sulfide junction - specifically, Cys, GSH, and to a lower extent homocysteine and methionine. Sulfide levels as the direct product of SIR activity, however, are not affected and kept constant, indicating the importance of sulfide homeostasis and a possible necessity for additional mechanisms to provide sulfide under these conditions, e.g., by desulfhydrase activity (Riemenschneider et al., 2005; Watanabe et al., 2008a; Álvarez et al., 2012; Kurmanbayeva et al., 2017). The metabolic signature downstream of the sulfite/sulfide junction constitutes a metabolite pattern resembling sulfate deprivation (Nikiforova et al., 2005). Such response patterns have also been reported in previous studies on SIR inhibition (Khan et al., 2010; Yarmolinsky et al., 2013; Yarmolinsky et al., 2014; Wang et al., 2016; Speiser et al., 2018), apart from the fact that thiols accumulated to higher levels in Arabidopsis knock-down plants (Khan et al., 2010). This deviating response might be explained by the fact that the Arabidopsis knock-down plants displayed a dwarf growth phenotype allowing the plant to accumulate metabolites while stalling growth, as observed for *Medicago truncatula* under combined phosphate and sulfate starvation with respect to GSH (Sieh et al., 2013). The resulting reduction of cysteine and other sulfur containing metabolites might be directly coupled to the observed dwarf phenotypes (Khan et al., 2010), as observed for other amino acid or nutrient imbalances (Hoefgen et al., 1995). Due to this “sulfur-starvation-like” metabolic phenotype, induction of the OAS cluster genes (Hubberten et al., 2012b) and, hence, a reduction of the glucosinolate content can be expected (Aarabi et al., 2016). Indeed, Khan et al. (2010) show a reduction of glucosinolates in *sir1-1.*

Further, the block in SIR activity resulted in the reduction of downstream sulfur containing organic compounds in the lines E2, P2, and P3. Such a reduction would resemble a response as described for sulfate starvation (Hirai et al., 2004), which, among other changes, resulted in SAM depletion (Nikiforova et al., 2005). SAM is not only a central methyl group donor but also a precursor of various compounds, such as polyamines, ethylene, phytosterols, nicotianamine, and others, and is hence involved in numerous cellular processes. Reduction of SAM assumingly would pleiotropically affect plant metabolism and growth (Roje, 2006). A more farfetched explanation might be an effect on iron sulfur cluster biogenesis due to imbalanced availability of reduced sulfur compounds (Balk and Pilon, 2011) in the SIR knock-down lines. This as well would massively affect plant metabolism in a pleiotropic manner (Couturier et al., 2013; Forieri et al., 2017) and would be reminiscent of sulfate starvation responses (Nikiforova et al., 2005).

Beyond that, the impairment of GSH contents will render the plants more sensitive to biotic and abiotic stresses as the GSH-ascorbate detoxification system will be impaired, as we showed experimentally by treating the plants with H_2_O_2_.

Upstream of the blockage of the sulfite/sulfide junction by *SIR* co-suppression, sulfite, sulfate, and thiosulfate accumulate (**Figures 3**, **4**), as has been shown previously (Khan et al., 2010; Brychkova et al., 2013; Yarmolinsky et al., 2014; Wang et al., 2016; Speiser et al., 2018). Interestingly, APR overproduction in Arabidopsis and maize also resulted in a similar pattern as overproduced sulfite was not accordingly converted to downstream reduced organic sulfur compounds, probably due to insufficient OAS availability (Tsakraklides et al., 2002; Martin et al., 2005). Hence, accumulating sulfite has to be detoxified. Unfortunately, no sulfate contents were determined, but as the downstream starvation signals are missing we would speculate that in these plants sulfate would not accumulate. Therefore, we suggest that the accumulation of upstream metabolites is due to a combination of signals from downstream metabolites, indicating a sulfur deficit and detoxification mechanisms to keep increasing sulfite contents at bay. OAS has been shown to induce the expression of Sulfate transporters (SULTRs), ATP sulfurylase, and APRs, while the reduced levels of GSH alleviate its negative control of SULTRs and APR (Hoefgen and Hesse, 2008; Takahashi et al., 2011). The respective metabolic and transcriptional changes have been shown in Arabidopsis *sir1-1* mutants (Khan et al., 2010; Speiser et al., 2018). Enzyme activities of SO (**Figure 2**), ATP sulfurylase, and APR were shown to be increased in tomato SIR RNAi plants (Yarmolinsky et al., 2014). Whether the reduced protein levels (**Figure 4**; Speiser et al., 2018) are due to protein degradation or reduced biosynthesis due to reduced cysteine and methionine availability cannot be decided here. Protein degradation would be a potential source for sulfide homeostasis through cysteine degradation by DES1 in a backward reaction of cysteine synthesis (**Figure 7**; Álvarez et al., 2012), or cyanide detoxification by cyano-alanine synthases (CAS) (Watanabe et al., 2008a) and rhodaneses (Most and Papenbrock, 2015).

Therefore, it can be concluded that the transgenic plants experience dual signals or metabolic compositions upon SIR impairment. On the one hand, downstream metabolite compositions resemble sulfate deprivation, thus triggering increased sulfate uptake and on the other hand, increased sulfite accumulation triggering detoxification mechanisms (**Figure 7**). Additional to sulfate starvation, induced uptake of sulfate APR expression and activity were shown to be increased (Yarmolinsky et al., 2014), which should theoretically result in sulfite accumulation. However, though sulfite levels increase with leaf age in impaired tobacco plants (**Figures 3**, **4**) sulfite contents remain comparably low. Only in older leaves a threshold seemed to be passed, which might cause the observed tissue damage (**Figure 1** and **Supplementary Figure 2A**). Sulfite is detoxified by a set of mechanisms (Brychkova et al., 2013, 2015). Peroxisomal SO converts sulfite to sulfate and H_2_O_2,_ the latter can further convert sulfite to sulfate in a non-enzymatic step (Hänsch et al., 2006). H_2_O_2_ - itself a potent toxic substance - is detoxified via ascorbate peroxidase (APX), which, however, is dependent on sufficient GSH for regeneration by glutathione reductase. As GSH levels are low in SIR impaired plants, H_2_O_2_ might accumulate. H_2_O_2_ can be further detoxified by superoxide dismutases and catalases (Dat et al., 2000). Sulfite can additionally be detoxified by the action of sulfite transferases (STR), i.e., GSH-S-transferases, probably STR1, and the sulfur dioxygenase ETHE1 (Papenbrock et al., 2011; Krüßel et al., 2014; Most and Papenbrock, 2015; Höfler et al., 2016; Speiser et al., 2018), which convert sulfite to thiosulfate (S_2_O_3_^2−^) using glutathione-persulfide (GSSH) to produce thiosulfate and GSH. Yarmolinsky et al. (2014) described an increase of sulfurtransferase activity and hence an increase in thiosulfate contents by increased sulfur transferase activity using 3-mercaptopyruvate (β-mercaptopyruvate) and sulfite. Thus, an alternative cysteine catabolic pathway *via* 3-mercaptopyruvate and persulfides is employed to detoxify sulfite to thiosulfate (**Figure 7**).

Thiosulfate is normally present only in low concentrations but is necessary for molybdenum cofactor biosynthesis by STR13 (CNX5) (Schwarz and Mendel, 2006) or is converted to rhodanid (SCN^−^) and sulfite by rhodaneses in order to detoxify cyanides (Papenbrock et al., 2011). As sulfite levels are increased, this might impair this detoxification reaction of CN^−^ and might contribute to tissue damage. β-cyano-alanine synthases might now be important to detoxify cyanides, although at the cost of cysteine, and the resulting β-cyano-alanine will be catabolized by a nitrilase (NIT4) to Asn and Asp (Piotrowski et al., 2001). However, sulfite, cyanide, and H_2_O_2_ are highly reactive compounds each exerting stress on the plant system. We therefore assume, as shown in **Figure 6**, that SIR co-suppression lines cannot tolerate additionally imposed stresses. This has also been shown for *SO* RNAi tomato lines, when internal sulfite levels were induced through dark exposition and additional ROS stress was imposed (Brychkova et al., 2013, 2015), and in Arabidopsis SIR knock-down lines treated with methylviologen, causing oxidative stress (Wang et al., 2016). Interestingly, in both systems, control plants react with increasing GSH contents in response to this stress, while SIR knock-down lines cannot respond accordingly due to the reduction of SIR activity.

Sulfite excesses might further be converted to increased amounts of sulfolipids (Brychkova et al., 2013). However, this does not appear to be a commonly applied pathway for sulfite detoxification. The expression of UDP-sulfoquinovose synthase was not altered in Arabidopsis *SIR* RNAi knock-down lines (Wang et al., 2016) or in tomato *SIR* RNAi lines (Yarmolinsky et al., 2014), while Khan et al. (2010) even reported a slight decrease of the expression of sulfolipid biosynthetic genes in the *SIR* knock-out, *sir1-1*. Despite this low effect on transcription a low but significant accumulation of sulfolipids in younger leaves but a decrease in older leaves was described for tomato RNAi lines (Yarmolinsky et al., 2014), linking the decrease of sulfolipids in older leaves rather to increasing senescence. This also fits to increased sulfite levels with increasing leaf age in tobacco co-suppression lines (**Figures 3**, **4**).

Detoxification mechanisms in all described plants with SIR activity impairment seem to run at the edge of their capacities. Externally applied stresses or internal stresses lead to tissue damages. As discussed, this might be the reason for the appearance of damage related phenotypes in older plant tissues or in parts of the leaf blades.

Imbalances in nutrient ion compositions affect metabolism of other nutrient ions (Hesse et al., 2004; Nikiforova et al., 2005; Astolfi et al., 2010; Sieh et al., 2013; Watanabe et al., 2013; Whitcomb et al., 2014; Zuchi et al., 2015). With respect to macronutrient anions, the increased sulfate contents resulted in increased nitrate and decreased phosphate levels. Nitrate accumulation under sulfate deprived condition has been described previously as both pathways are closely linked. Though it has been shown that reduced sulfate availability negatively affects nitrate uptake and nitrate reduction (Kopriva and Rennenberg, 2004) accumulation of nitrate and N-rich organic compounds still does occur (Nikiforova et al., 2005), probably due to a complex combination of breakdown of proteins and other compounds to retrieve sulfur, imbalances in amino acid biosynthesis (Nikiforova et al., 2006). Again, this response indicates a role of metabolites downstream from sulfite as inducers of a sulfate starvation like response despite the availability of sulfate in the SIR co-suppression mutants. Further, one can thus infer that high sulfate and nitrate levels directly affect the phosphate uptake system and that downstream metabolites are not involved, as these display a sulfate starvation pattern, which should result rather in P increases. As sulfide levels are constant, sulfide might not be involved in regulating nutrient ion homeostasis.

It can though not be excluded with this experimental setup that GSH might exert a regulatory function. Reduction of GSH levels might affect in a systemic manner regulation of root sulfate uptake. In addition, metabolite levels in roots were not investigated in this study. Previous experiments under sulfate starved conditions using split root and separated agar systems indicated a local *in situ* control of sulfate uptake at roots, but high leaf sulfate status negatively affected sulfate starvation response genes in the root (Hubberten et al., 2012b) or local supply of GSH.

Chlorophyll contents and photosynthetic activity are reduced as described, with chlorophyll b being less affected, as indicated by the decreased chlorophyll a/b ratio (**Figure 5B**). One possible explanation is that chlorophyll methylation might be impaired due to the assumed depletion of the methyl group donor *S*-adenosyl-L-methionine (SAM). In detail, partial SIR silencing would thus affect the conversion of Mg-protoporphyrin IX to Mg-protoporphyrin IX monomethyl ester, catalyzed by MgP methyltransferase (Tanaka and Tanaka, 2007). However, young leaves accumulate similar levels of chlorophyll, arguing against a general defect in chlorophyll biosynthesis. Furthermore, most photosynthetic complexes such as light harvesting proteins, photosystem I and the cytochrome b_6_f complex (and the chlorophylls they contain) are believed to be long-lived, with halftimes in the range of several days to weeks (Hojka et al., 2014; Nelson et al., 2014; Li et al., 2017). Therefore, it is unlikely that the defects in chlorophyll accumulation and photosynthetic electron transport can be explained by an impairment of chlorophyll biosynthesis only occurring in mature leaves. Only the PSII reaction center, especially the D1 protein, undergoes a much more rapid turn-over. D1 is oxidatively damaged by singlet oxygen produced as a side-product of PSII photochemistry and depending on the light intensity may be exchanged between once per day and once per hour (Aro et al., 1993; He and Chow, 2003). Here, the clear PSII photoinhibition observed in mature leaves of the strong co-suppression line (**Figure 5E**) indicates that such a situation of high PSII reaction center turn-over exists in the co-suppression mutants. However, this process alone is unlikely to account for the accelerated loss of chlorophyll in the co-suppression lines.

Instead, chlorophyll content reduction might be caused by direct deleterious sulfite reactions with chlorophylls (Murray, 1997; Brychkova et al., 2007). Alternatively, leaf senescence pathways might be triggered prematurely in the co-suppression lines, possibly due to increased oxidative damage to PSII and increased production of reactive oxygen species such as singlet oxygen. Especially singlet oxygen is known to trigger either a cell death program (Kim et al., 2012), which could explain the necrotic leaf segments, or to accelerate leaf-senescence associated pathways in a dose-dependent way (Krieger-Liszkay et al., 2015; Nakamura and Izumi, 2018). Activation of leaf senescence pathways could then induce the degradation of the entire photosynthetic apparatus, possibly via chlorophagy (Nakamura and Izumi, 2018), resulting in the observed accelerated loss of total chlorophyll (**Figure 5C**) and PSI (**Figure 5F**).

The observed chlorophyll reduction and reduced photosynthetic electron transport capacity (**Figure 5H**) might be one reason for the reduced concentrations of photosynthetic metabolites. Fructose, glucose, sucrose, and starch contents are all reduced in comparison to WT (**Figure 4**). This is consistent with previous studies on Arabidopsis being exposed to sulfate starvation under normal light conditions (Wulff-Zottele et al., 2010).

The imbalance in certain amino acids, here the sulfur containing amino acids Cys and Met, leads to the response of over-accumulation of other amino acids, as described previously (Hoefgen et al., 1995). The sulfur starvation signals exhibited by the SIR blockage together with impairment of photosynthetic capacity resulted in a profound reduction of protein contents and increase of amino acids (Wulff-Zottele et al., 2010). As discussed, protein degradation would be a potential source for sulfide homeostasis through cysteine degradation (Watanabe et al., 2008a; Álvarez et al., 2012; Most and Papenbrock, 2015). Especially, the observed low contents of proline (**Figure 4**) are counter-intuitive, as presumed stress by sulfite intoxication and detoxification mechanisms potentially resulting in H_2_O_2_ could be assumed to induce proline accumulation, usually expected as stress marker. However, reduced proline contents under sulfate deprivation have been determined before (Wulff-Zottele et al., 2010). In that study proline accumulation occurred only under the dual stress of sulfate starvation and high-light exposure.

This analysis provides beyond the described metabolic consequences of SIR activity impairment information with respect to the question, how sulfate metabolism is regulated under sulfate limiting conditions (Saito, 2004; Davidian and Kopriva, 2010; Hubberten et al., 2012b). The combination of sulfate accumulation and induction of SULTRs and APR (Yarmolinsky et al., 2014) together with the typical metabolic signature for sulfate deprived plants (Nikiforova et al., 2005), i.e., OAS increase and cysteine, methionine, and GSH decrease, speak for the fact that either of these metabolites has a signaling function (Kopriva, 2006; Hubberten et al., 2012b; Aarabi et al., 2016). As sulfide levels remained constant, we do not assume a signaling function of sulfide on the sulfate uptake and assimilation system. Accumulating sulfate, whose tissue levels are normally strictly controlled, indicates that the downstream starvation signals override any signal from sulfate to the uptake system. Thus, it remains unclear, how plants “measure” cytosolic and vacuolar sulfate concentrations in leaves and how leave and root tissues communicate, as has been discussed previously (Hubberten et al., 2012a). A specific investigation of the root system would be necessary to further elucidate this question. The sulfite/sulfide junction is indeed crucial for plant performance, as the existing detoxification systems (oxidation to sulfate, conversion to thiosulfate or sulfolipids) are insufficient to cope with inhibitions of SIR activity below certain thresholds, which could be demonstrated by further stressing the plants. However, it cannot be decided here whether the GSH depletion or the accumulation of reactive compounds such as either sulfite, cyanide, or H_2_O_2_ are causative for the damage or a combination of all. Either SIR activity cannot be reduced further to still allow sufficient sulfide biosynthesis and/or reverting activities providing GSH and sulfide (DES1; Álvarez et al., 2012) are necessary to allow plant survival. In tobacco SIR co-suppression lines, plants actually invest in keeping sulfide contents constant and comparable to WT, while downstream metabolites are reduced. Thus, sulfide homeostasis can be speculated to be sensitive and to play a crucial role in plant metabolism.

## Materials and Methods

### Plant Materials

The investigated plants were derived from a large-scale project with transgenic tobacco plants (*Nicotiana tabacum* L., cv. Samsun NN) (Lein et al., 2008). The plants were selected by phenotypic traits (chlorotic and necrotic phenotypes). It was found that the transcription of SIR was impaired by co-suppression in the plants (**Supplementary Figure 1**). Several independent co-suppression lines of SIR were prepared (Lein et al., 2008). The plants chosen are construct numbers E-18042 (termed E2), P-14921 (termed P2), and P-14921-2_3a (termed P3). No obvious differences between investigated co-suppression lines of SIR and the WT in plant height and biomass (fresh weight) were observed. Plants were cultured on soil under standard conditions in a greenhouse (140 μmol m^−2^ s^−1^, 50% humidity, 21°C) at a 16 h light/8 h dark cycle. Samples were immediately frozen in liquid nitrogen and stored at −80°C until further use. Five biological replicates were used for all the analyses described in this study.

### Chemical Abbreviations

HEPES, 4-(2-hydroxyethyl)-1-piperazineethanesulfonic acid; EDTA, ethylenediaminetetraacetic acid; EGTA, ethylene glycol tetraacetic acid; DTT, dithiothreitol; PMSF, phenylmethylsulfonyl fluoride; Tris, tris(hydroxymethyl)aminomethane; ddH_2_O, double-distilled water; CHES, *N*-cyclohexyl-2-aminoethanesulfonic acid; NADP ^+^/NADPH, nicotinamide adenine dinucleotide phosphate (oxidized/reduced form).

### Sulfite Reductase (SIR) Activity

Frozen material was homogenized and 100 mg was dissolved in 0.5 mL of extraction buffer (50 mM HEPES/KOH, pH 7.5, 10 mM KCl, 10 mM EDTA, 1 mM EGTA, 10% glycerol, 10 mM DTT, 0.5 mM PMSF). After centrifugation at 4°C and 20,000 *g* for 10 min two times to remove insoluble components, the supernatant was placed on a NAP5 column (GE Healthcare). The column was previously equilibrated with a buffer (50 mM HEPES/KOH, pH 7.5, 1 mM EDTA). After applying the supernatant, the flow was discarded. Subsequently, 1 mL of resuspension buffer (50 mM HEPES/KOH, pH 7.5, 1 mM EDTA, 0.5 mM PMSF, and 2 mM DTT) was added to the column and the resulting flow was collected. For measurement of SIR activity, the flow (60 μL) was reacted with 160 μL of master mix (25 mM HEPES, pH 7.5, 1 mM Na_2_SO_3_, 5 mM *O*-acetylserine, 1 μL of *O*-acetylserine(thiol)lyase enzyme, 10 mM DTT, 30 mM NaHCO_3_, 15 mM Na_2_S_2_O_4_, 5 mM methyl viologen) for 1 h at RT in the dark. The reaction was stopped by adding 100 μL of 20% trichloroacetic acid (TCA). After centrifugation at 4°C and 20,000 *g* for 3 min, cysteine produced in the supernatant (250 μL) was detected by a ninhydrin reaction with 300 μL of ninhydrin solution (250 mg of ninhydrin dissolved in 6 mL of acetic acid and 4 mL of 12 N HCl) and 200 μL of acetic acid at 99°C for 10 min. The solution was then left to cool at RT for 10 min and an optical density of 560 nm was measured.

### Sulfite Oxidase (SO) Activity

Frozen material was homogenized and 100 mg was dissolved in 400 μL of 0.1 M Tris-acetate buffer (pH 7.25). The extract was sonicated three times and then centrifuged at 4°C and 20,000 *g* for 20 min. The supernatant was mixed with 1 mL of ice-cold saturated ammonium sulfate solution and centrifuged at 4°C and 20,000 *g* for 15 min. The supernatant was discarded and the pellet was dissolved in 200–400 μL of the Tris-acetate buffer. The concentration of protein was determined by Bradford assay (Bradford, 1976) and 50 μg of protein was used for SO activity assay. The 50 μg of protein was dissolved in a total volume of 400 μL with 0.1 M Tris-acetate buffer. The reaction was started by adding 100 μL of 0.5 mM sulfite with 900 μL of sulfite detection reagent (100 μL of reagent A; dissolve 400 mg of fuchsin in 125 ml of concentrated sulfuric acid and make up to 1 L of ddH_2_O, 100 μL reagent B; 3.2% formaldehyde, and 700 μL of ddH_2_O). The reaction was stopped immediately after the addition of sulfite (T0), after 10 min (T1), 20 min (T2), and 30 min (T3). The optical density of the solution was measured at 560 nm.

### Determination of OAS and Amino Acid Contents

*O*-acetylserine and amino acids contents were determined following a protocol modified from Hubberten et al. (2012b). Frozen ground material (100 mg) was homogenized in 400 μL of 80% (v/v) aqueous ethanol (buffered with 2.5 mM HEPES/KOH, pH 6.2 for OAS or pH 7.5 for amino acids, respectively), 400 μL of 50% (v/v) aqueous ethanol (buffered with 2.5 mM HEPES/KOH, pH 6.2 or pH 7.5), and 200 μL of 80% (v/v) aqueous ethanol. All three combined supernatant fractions were derivatized with *O*-phthalaldehyde and then subjected to high performance liquid chromatography (HPLC) analysis with fluorescence detection (Dionex) using a Hyperclone C18 (ODS; octadecylsilane) column (Phenomenex). OAS and amino acids were eluted with an increasing methanol/acetonitrile gradient comprising buffer A (1% v/v tetrahydrofolate, 8.5 mM sodium phosphate buffer, pH 6.2 for OAS or 0.2% v/v tetrahydrofolate, 8.5 mM sodium phosphate buffer, pH 6.8 for amino acids, respectively) and buffer B (32.5% v/v methanol, 20.5% v/v acetonitrile, 18.5 mM sodium phosphate buffer, pH 6.2 or pH 6.8), as described by Hubberten et al. (2012b).

### Determination of Thiol Contents

Thiols were determined following a protocol modified from Hubberten et al. (2012b). Frozen ground material (50 mg) was homogenized in 1 mL of 0.1 M HCl and 30–50 mg of polyvinylpolypyrrolidone (PVPP) washed with 0.1 M HCl and shaken (1,000 rpm) for 45 min at room temperature (RT). After centrifugation at 4°C and 20,000 *g* for 20 min, the supernatant was taken and immediately used for the reduction step. Thiols in the extract (120 μL) were reduced with 70 μl of 10 mM DTT and 200 μl of 0.25 M CHES-NaOH buffer, pH 9.4 for 45 min at RT. Derivatization was carried out with 10 μL of 25 mM monobromobimane for 15 min at RT in the dark. The reaction was stopped by adding 220 μL of 100 mM methanesulfonic acid. After centrifugation at 4°C and 20,000 *g* for 30 min, the supernatants were subjected to HPLC analysis with fluorescence detection (Dionex) using a C18 column (Knauer, Berlin, Germany). Thiols were eluted with an increasing methanol gradient comprising buffer A (0.5% acetic acid, pH 4.0) and buffer B (100% methanol), as described by Hubberten et al. (2012b).

### Determination of Anion Contents

Frozen ground material (50 mg) was homogenized in 500 μL of 0.1 mM HCl. Samples were centrifuged at 4°C and 20,000 *g* for 5 min. The supernatant was transferred to an Ultrafree MC 5000 MC NMWL Filter Unit (Millipore) and centrifuged at 5,000 *g* and 4°C for 90 min. After filtration, samples were diluted 20 times with deionized water and analyzed using the Dionex ICS-2000 system with a KOH gradient, following manufacturer’s protocol (Dionex).

### Determination of Further Metabolites by a 96-Well Platform

Frozen ground material (20 mg) was extracted twice with 250 μL and 150 μL of 80% (v/v) aqueous ethanol (buffered with 2 mM HEPES, pH 7.5) for 30 min at 95°C and once with 250 μL of 50% (v/v) aqueous ethanol for 30 min at 95°C. All three supernatant fractions were combined. All assays were prepared in 96-well polystyrene microplates using a JANUS automated workstation robot (Perkin-Elmer, Zaventem, Belgium), according to established protocols (Gibon et al., 2004; Cross et al., 2006). Total free amino acids were assayed using fluorescamine (Bantan-Polak et al., 2001). Proline was quantified using ninhydrin assay (Bates et al., 1973). Glucose, fructose, and sucrose were quantified based on the conversion of NADP^+^ to NADPH by hexokinase and glucose-6-phosphate dehydrogenase, phosphoglucoisomerase, and invertase, which convert NADP^+^ to NADPH proportional to the glucose units. NADPH was quantified by the absorption of light at 340 nm (Stitt et al., 1989). Proteins were extracted from the pellet with 400 μL of 100 mM NaOH for 30 min at 95°C (Hendriks et al., 2003). The concentration of protein was determined by Bradford assay (Bradford, 1976). After neutralization, starch was digested to glucose by amyloglucosidase and amylase at 37°C for 16 h. Determination of the glucose was assayed enzymatically by coupling to reduction of NADP^+^ to NADPH (Stitt et al., 1989).

### Photosynthetic Parameters

Leaf absorptance was measured using an integrating sphere (ISV-722) attached to the V-650 spectrophotometer (Jasco Deutschland GmbH, Pfungstadt, Germany). The spectral bandwidth was set to 1 nm, and the scanning speed was 200 nm min^−1^. Transmittance and reflectance spectra were measured between 750 and 400 nm wavelength, and leaf absorptance was calculated as 100% – transmittance (%) – reflectance (%). Then, the average leaf absorptance was calculated for the range of photosynthetically active radiation (400–700 nm wavelength). Chlorophyll a fluorescence of intact leaves was measured at room temperature using a Dual-PAM-100 instrument (Heinz Walz). Light-response curves of linear electron transport were recorded on intact leaves after 30 min of dark adaptation. First, the maximum quantum efficiency of PSII in the dark-adapted state (F_V_/F_M_) was determined. Then, the amount of redox-active PSI (Pm) was determined by illumination of the dark-adapted leaf disk with far-red light for 8 s to selectively oxidize PSI and the high-potential chain, followed by the application of a saturating light pulse (5000 μE m^−2^ s^−1^, 600 ms duration), to activate linear electron flux and fully reduce PSI again. Afterward, actinic light intensity was increased in 20 steps up to 2000 μE m^−2^ s^−1^, with measuring intervals per light intensity between 150 s under light-limited conditions and 60 s under light-saturated conditions. At the end of each step, a saturating light pulse was applied (5000 μE m^−2^ s^−1^, 600 ms duration), and the effective PSII quantum yield was used to calculate linear electron transport, using the leaf absorptance value previously determined in the integrating sphere. Finally, the chlorophyll content of the measured leaf disks was determined according to Porra et al. (1989) in 80% (v/v) acetone.

## Author Contributions

MN and H-MH: experimental design, performing the experiments, and data interpretation. MW: data interpretation and preparing the figures. RoH: support with enzyme activity determinations. MS: measuring photosynthetic parameters and data interpretation. RaH: experimental design, data interpretation, and manuscript preparation. All the authors discussed the results and commented on the manuscript.

## Conflict of Interest Statement

The authors declare that the research was conducted in the absence of any commercial or financial relationships that could be construed as a potential conflict of interest.
