# Supplementary material for: Sulfite Reductase Co-suppression in Tobacco Reveals Detoxification Mechanisms and Downstream Responses Comparable to Sulfate Starvation
Source: Front Plant Sci. 2018 Oct 15;9:1423. doi: 10.3389/fpls.2018.01423 (PMC6196246; doi:10.3389/fpls.2018.01423)
Supplement: Supplementary file 1 [file Data_Sheet_1.PDF]

```
               1       10        20        30        40        50        60        70        80        90        100       110       120       130
NtSIR          MTTSFGAAINIAVADDPNPKLQIHNFSGLKSTSNSLLLSRRLHVFQSFSPSNPSSIVRAVSTPAKPAAVEPKRSKVEIFKEQSNFIRYPLNEEILNDAPNINEAATQLIKFHGSYMQYDRDERGGRSYSF
Transformation
Consensus      ..................................................................................................................................

               131     140       150       160       170       180       190       200       210       220       230       240       250       260
NtSIR          MLRTKNPGGEVPNRLYLVMDDLADQFGIGTLRLTTRQTFQLHGVLKKNLKTVMSTIIKNMGSTLGACGDLNRNVLAPAAPFAKKDYMFAKQTADNIAALLTPQSGFYYDVWVDGEKVMTAEPPEVVKARN
Transformation
Consensus      ..................................................................................................................................

               261     270       280       290       300       310       320       330       340       350       360       370       380       390
NtSIR          DNSHGTNFPDSPEPIYGTQFLPRKFKIAVTVPTDNSVDIFTNDIGVVVVSNEDGEPQGFNIYVGGGMGRTHRMETTFPRLAEPLGYVPKEDILYAVKAIVVTQRENGRRDDRRYSRLKYLLSSWGIEKFR
Transformation
Consensus      ..................................................................................................................................

               391     400       410       420       430       440       450       460       470       480       490       500       510       520
NtSIR          SVTEQYYGKKFQPCRELPEWEFKSYLGWHEAGDGSLFCGLHVDNGRVKGAMKKALREVIEKYNLNVRLTPNQNIILCNIRQAWKRPITTVLAQGGLLQPRYVDPLNLTAMACPAFPLCPLAITEAERGIP
Transformation                                                                                                                                 GIP
Consensus      ...............................................................................................................................GIP

               521     530       540       550       560       570       580       590       600       610       620       630       640       650
NtSIR          DILKRVRAIFERVGLKYSESVVIRITGCPNGCARPYMAELGLVGDGPNSYQIWLGGTPNQTSLAKTFKDKLKVQDLEKVLEPLFFHWRRKRQSKESFGDFTNRMGFEKLGEFVEKWEGIPESSSRYNLKL
Transformation DILKRVRAIFEKVGLKYSESVVIRVTGCPNGCARPYMAELGLVGDGPNSYQIWLGGTPNQTSLAKTFKDKVKVQDLEKVLEPLFFHWRRKRQSKESFGDFTNRMGFEKLGEFVEKWEGIPESSSRYNLKL
Consensus      DILKRVRAIFE.VGLKYSESVVIR!TGCPNGCARPYMAELGLVGDGPNSYQIWLGGTPNQTSLAKTFKDK.KVQDLEKVLEPLFFHWRRKRQSKESFGDFTNRMGFEKLGEFVEKWEGIPESSSRYNLKL

               651     660       670       680       690693
NtSIR          FADRETYEAMDALASIQDKNAHQLAIEVVRNYVASQQNGKSMD
Transformation FADRETYEAMDALASIQDKNAHQLAIEVVRNYVASQQNGKSMD
Consensus      FADRETYEAMDALASIQDKNAHQLAIEVVRNYVASQQNGKSMD
```

Supplemental Figure 1. Amino acid sequence of SIR protein for the transformation. Alignment of the amino acid sequence of the tobacco SIR protein against the sequence used for the transformation. The complete sequence of SIR protein consists of 693 amino acids and the transformed sequence consists of 176 amino acids.
