# Supplementary material for: Sulfite Reductase Co-suppression in Tobacco Reveals Detoxification Mechanisms and Downstream Responses Comparable to Sulfate Starvation
Source: Front Plant Sci. 2018 Oct 15;9:1423. doi: 10.3389/fpls.2018.01423 (PMC6196246; doi:10.3389/fpls.2018.01423)
Supplement: Supplementary file 2 [file Data_Sheet_2.PDF]

(A)

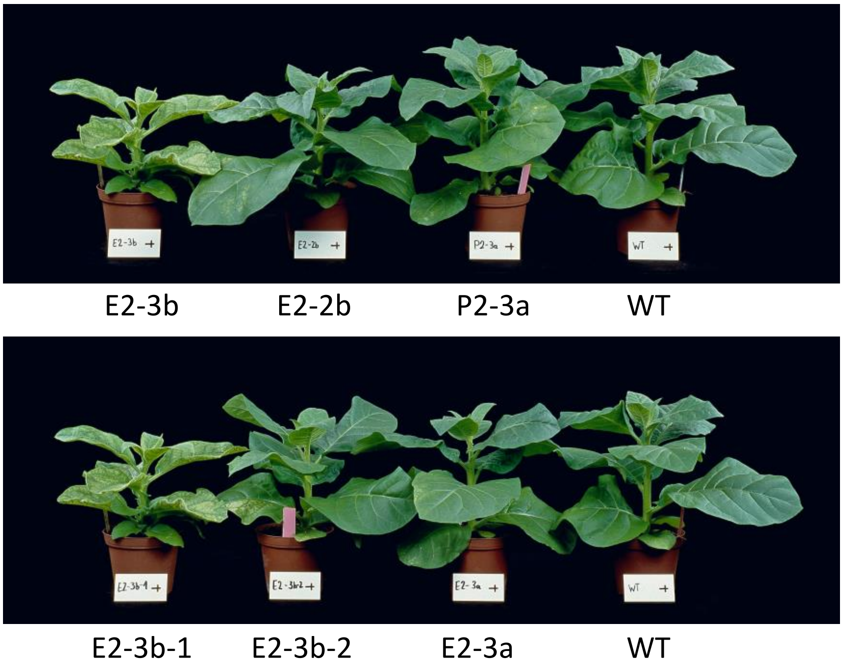

(B)

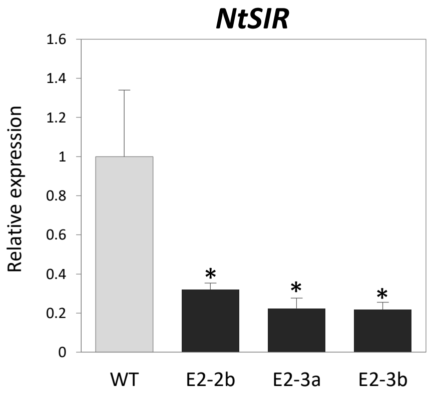

(C)

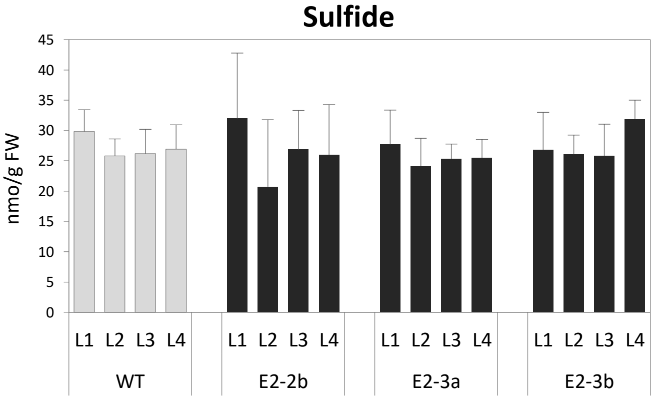

Supplemental Figure 2. *SIR* expressions and sulfide contents in SIR co-suppression tobacco lines. (A) SIR co-suppression tobacco lines at the harvesting stage. Each panel shows a representative plant. (B) *NtSIR* expression in leaves (L4). Data represent the mean (±SD) of four biological replicates. (C) Sulfide contents. Data represent the mean (±SD) of three biological replicates. Differences between wild-type plants and transgenic lines were analyzed using Student's *t* test and statistical significance was indicated (*, $P < 0.05$).

Materials and Methods
Supplemental Figure 2:
Plant materials

Plants were cultured on soil under standard conditions in a greenhouse (140 µmol m^{-2} s^{-1}, 50% humidity, 21° C) at a 16 h light/8 h dark cycle. Samples were immediately frozen in liquid nitrogen and stored at -80° C until further use.

Gene expression analysis of *SIR* gene

RNA was extracted from leaves (L4) using an RNeasy plant mini kit (Qiagen; http://www.qiagen.com/) according to the manufacturer’s instructions. Total RNA was digested with Turbo DNAfree™ DNase (Ambion). A 1 µg aliquot of total RNA was subjected to cDNA synthesis using Superscript III reverse transcriptase (Invitrogen; http://www.invitrogen.com/). PCR was performed in a 384-well plate using an ABI PRISM® 7900 HT sequence detection system (Applied Biosystems; http://www.appliedbiosystems.com/). Reactions contained 5 µl of 2x SYBR® Green Master Mix reagent (Applied Biosystems), 1 µl of cDNA and 200 nM of each gene-specific primer. SDS 2.0 software (Applied Biosystems) was used for data analysis. C_T values for genes were normalized to the C_T values for elongation factor 1 (EF1). Primer sequences: SIR_forward primer, GATTAACATCGCCGTCGCCG; SIR_reverse primer, CGCCTGCTAAGCAACAGCGA; EF1_forward primer, TGAGCACGCTCTTCTTGCTTTCA; EF1_reverse primer, GGTGGTGGCATCCATCTTGTTACA.

Determination of sulfide contents

Fifty mg of homogenized plant tissue were incubated for 2-3 min in 200 µl of 0.1 M HCl. Then the thiols were derivatized by adding 300 µl of 0.25 M 2-(cyclohexylamino)ethanesulfonic acid (CHES)/NaOH, pH 9.4, and 20 µl of 25 mM monobromobimane. The reaction was performed in the dark at 4° C for 15 min, and stopped by adding 200 µl of 65 mM methane sulfonic acid. The samples were subjected to HPLC analysis using a LiChrospher® 60 RP-select B (5 µm) LiChroCART® 125-4 chromatography column in a Dionex Summit HPLC system. Thiols were eluted with an increasing methanol gradient comprising buffer A (0.25 M acetic acid, pH 4.25) and buffer B (100% methanol) as described in Hubberten et al. (2012). Measured substances were validated by pre-incubation of the extract with 2,2′-dithiopyridine and comparison to standard substances.

Reference: Hubberten H. M., Klie S., Caldana C., Degenkolbe T., Willmitzer L., Hoefgen R. (2012). Additional role of *O*-acetylserine as a sulfur status-independent regulator during plant growth. Plant J. 70, 666-677.
